# A Systematic Review of Tafamidis in Patients With Transthyretin Amyloid Cardiomyopathy

**DOI:** 10.7759/cureus.18221

**Published:** 2021-09-23

**Authors:** Bishnu Mohan Singh, Narayan Bohara, Kamal Gautam, Madan Basnet, Sistu KC, Binod KC, Anuradha Raut, Abisha Phudong, Jeevan Gautam

**Affiliations:** 1 Division of Clinical and Translational Research, Larkin Community Hospital, Miami, USA; 2 Emergency and General Medicine, Patan Academy of Health Sciences, Lalitpur, NPL; 3 Cardiology, Oxford University Clinical Research Unit, Patan Academy of Health Sciences, Lalitpur, NPL; 4 Internal Medicine, Oxford University Clinical Research Unit, Patan Academy of Health Sciences, Lalitpur, NPL; 5 Medicine, Nepalese Army Institute of Health Sciences, Kathmandu, NPL; 6 Internal Medicine, Patan Academy of Health Sciences, Lalitpur, NPL; 7 Internal Medicine, Upstate University Hospital, New York, USA; 8 Internal Medicine, Nepal Medical College and Teaching Hospital, Kathmandu, NPL; 9 Emergency Department, Care and Cure Multispeciality Hospital, Lalitpur, NPL; 10 Internal Medicine, Tribhuvan University Teaching Hospital, Kathmandu, NPL

**Keywords:** randomized controlled trial, cardiomyopathies, amyloid, ttr-stabilizing drug, cardiac biomarkers, echocardiography, outcomes, heart failure, tafamidis, transthyretin amyloid cardiomyopathy

## Abstract

Transthyretin amyloid cardiomyopathy disease burden is increasing daily due to advancements in diagnostic and imaging modalities in the modern world. Tafamidis is one of many therapeutic options. The main objective of this review is to study the role of Tafamidis in slowing the progression of transthyretin cardiomyopathy (TTR-CM) by analyzing randomized controlled trials (RCTs) and non-RCTs of Tafamidis. We searched for published papers of Tafamidis in the English language in electronic databases like Google Scholar, PubMed, Cochrane Library, and PubMed Central. We imported the resulting articles from our search to Mendeley software. Four reviewers removed the duplicates and performed title and abstract screening of the articles. The same reviewers obtained the full-text of relevant articles and did full-text screening based on eligibility criteria. Finally, five reviewers performed a quality assessment of RCTs using the Cochrane risk of bias assessment and of non-RCTs by a checklist prepared by Downs and Black. Any disagreements about any process were resolved by a discussion with other authors. One RCT and five non-RCTs of Tafamidis were included in this systematic review. From the non-RCTs, stability was observed in different parameters like echocardiographic findings, cardiac biomarkers, and ECG in patients with transthyretin cardiomyopathy during the study duration with Tafamidis. ATTR-ACT (Tafamidis in Transthyretin Cardiomyopathy Clinical Trial) trial demonstrated reduction of cardiovascular events and all-cause mortality in the Tafamidis group in comparison to placebo. In both RCT and non-RCTs, Tafamidis was established as a safe and tolerable drug for patients with TTR-CM. Our study proved the role of Tafamidis in reducing cardiovascular events, all-cause mortality, and the progression of cardiac disease in TTR-CM patients. In addition to five non-RCTs, current evidence is based on the findings of only one RCT of Tafamidis. Hence, evidence from additional RCTs is required to strongly support the stability of parameters like echocardiographic findings, cardiac biomarkers, and ECG with Tafamidis use.

## Introduction and background

Amyloidosis refers to a group of heterogeneous diseases caused by the extracellular tissue deposition of fibrils composed of abnormally folded proteins. It is a multi-systemic disease affecting various organs, including the heart, kidney, liver, lungs, skin, central and peripheral nervous systems, muscles, skin, etc. Although there are more than 30 proteins causing amyloid deposition in various systems [1], cardiac involvement is mainly caused by misfolded transthyretin (ATTR) or immunoglobulin light chain (AL) depositions [2]. ATTR amyloidosis can occur due to normal wild-type amyloidogenic TTR (transthyretin) protein or due to a variant of the TTR gene (hereditary ATTR amyloidosis, ATTRv). Cardiac involvement can cause diastolic or systolic dysfunction, arrhythmias, heart block, or infarctions due to amyloid deposition in coronary vessels.

Transthyretin amyloid cardiomyopathy is often an under-diagnosed cause of heart failure, especially in the older population [3]. It usually presents with signs and symptoms of heart failure like dyspnea, syncope, fatigue, and orthostatic hypotension. It is a rapidly progressive disease, with a median survival of untreated cases of wild-type ATTR as low as 3.6 years [4]. The mainstay of treatment is symptomatic care of heart failure, which is more beneficial in patients with ATTRm (familial transthyretin-associated) and ATTRwt (wild-type transthyretin-associated) amyloidosis. Surgical treatment options like heart transplantation, liver transplantation to remove mutant TTR from blood may be helpful to some patients [5-6]. Patisiran and Inotersen are RNA-targeted therapies approved by the US Food and Drug Administration (FDA) for transthyretin-mediated hereditary amyloidosis polyneuropathy but are emerging therapies for transthyretin cardiomyopathy (TTR-CM) [7]. In contrast, Tafamidis (TTR stabilizer) is an FDA-approved drug for TTR-CM patients. Although a few clinical studies have shown a beneficial effect of Tafamidis in slowing the progression of amyloid cardiomyopathy, a thorough study comparing the overall effects of Tafamidis is still lacking. In this study, we aim to examine the effectiveness of Tafamidis in slowing the progression of cardiomyopathy and its safety profile, effect on various cardiac parameters, and overall improvement of symptoms by evaluating existing clinical studies.

## Review

Methods

We followed the guidelines of the Preferred Reporting Items for Systematic reviews and Meta-Analyses (PRISMA 2020) for conducting the systematic review [8].

Study Protocol

We performed preliminary searches and literature reviews on our research question. We then prepared our protocol according to the guidelines of the Preferred Reporting Items for Systematic Review and Meta-Analysis Protocols 2015 (PRISMA-P 2015) [9]. We published our protocol in the Research Registry, with a unique identifying number: reviewregistry1177.

Search Strategy

We used electronic databases like PubMed, Cochrane Library, PubMed Central (PMC), and Google Scholar for searching relevant articles to answer our research question until May 2021. We customized our search to include any clinical studies that had evaluated Tafamidis’s role in treating transthyretin amyloid cardiomyopathy. We searched for English language studies conducted in human subjects. The search strategies for different electronic databases are shown in Appendices.

Study Selection

After we completed our search, we imported all articles into Mendeley software. Then, we removed the duplicates in the Mendeley software. Four reviewers (N. B., K.G., B.M.S., and S.K.) independently screened the articles based on their titles and abstracts. The same reviewers again did full-text screening independently. All conflicts were resolved by discussion with other authors. At every step, we used our eligibility criteria to screen and finally selected the studies included in our systematic review. The inclusion criteria were as follows: (i) randomized controlled trials (RCTs), non-RCTs, or single-arm studies of Tafamidis that included patients with transthyretin amyloid cardiomyopathy, either wild-type or variant-type; (ii) studies done on patients of any race and age 18 years or above; (iii) studies with the following outcomes: TTR stabilization, cardiac biomarkers, ECG or Holter parameters, echocardiographic parameters, functional tests, quality of life, arrhythmias, hospital admission, and death; (iv) studies published in the English language. The exclusion criteria were as follows: (i) systematic reviews, meta-analyses, case reports, and case series; (ii) studies done on healthy volunteers only, the pediatric population (<18 years), diseases other than transthyretin amyloid cardiomyopathy; (iii) studies with only non-cardiovascular outcomes; (iv) studies published in a non-English language.

Data Extraction

We extracted the following data from the studies included in our systematic review: (i) participants in the Tafamidis group and the control group; (ii) duration of follow-up; (iii) participant characteristics like mean age, sex, mean body mass index (BMI), and race; (iv) the dose of Tafamidis; (v) target population; (vi) study design; (vii) adverse events noted among participants; (viii) changes in the level of cardiac biomarkers during the study period; (ix) all-cause mortality; (x) cardiovascular-related hospitalization; (xi) ambulatory changes; and (xii) quality of life assessments during the study duration. Five reviewers (N.B., K.G., M.B., J.G., B.K., and A.R.) extracted the data independently, and any conflicts during the process of data extraction were resolved by discussion with other authors.

Quality Assessment

We assessed the quality of the included studies by using the Cochrane quality assessment tool for RCTs [10], and a quality assessment of the included non‐RCTs was performed using a checklist by Downs and Black [11]. We analyzed the risk of bias in RCT using the risk of bias 2 (RoB 2) tool under the following headings: random sequence generation (selection bias), allocation sequence concealment (selection bias), blinding of participants and personnel (performance bias), blinding of outcome assessment (detection bias), incomplete outcome data (attrition bias), selective outcome reporting (reporting bias), and other potential sources of bias. Depending on the risk of bias, the tool rated RCT as “Low risk,” “Unclear risk,” and “High risk.” Five reviewers (B. M. S., K.G., M.B., N.B., and A.P.) independently assessed the risk of bias of included studies. Any disagreements were resolved by discussion with other authors.

Results

Literature Selection

**Figure 1:**
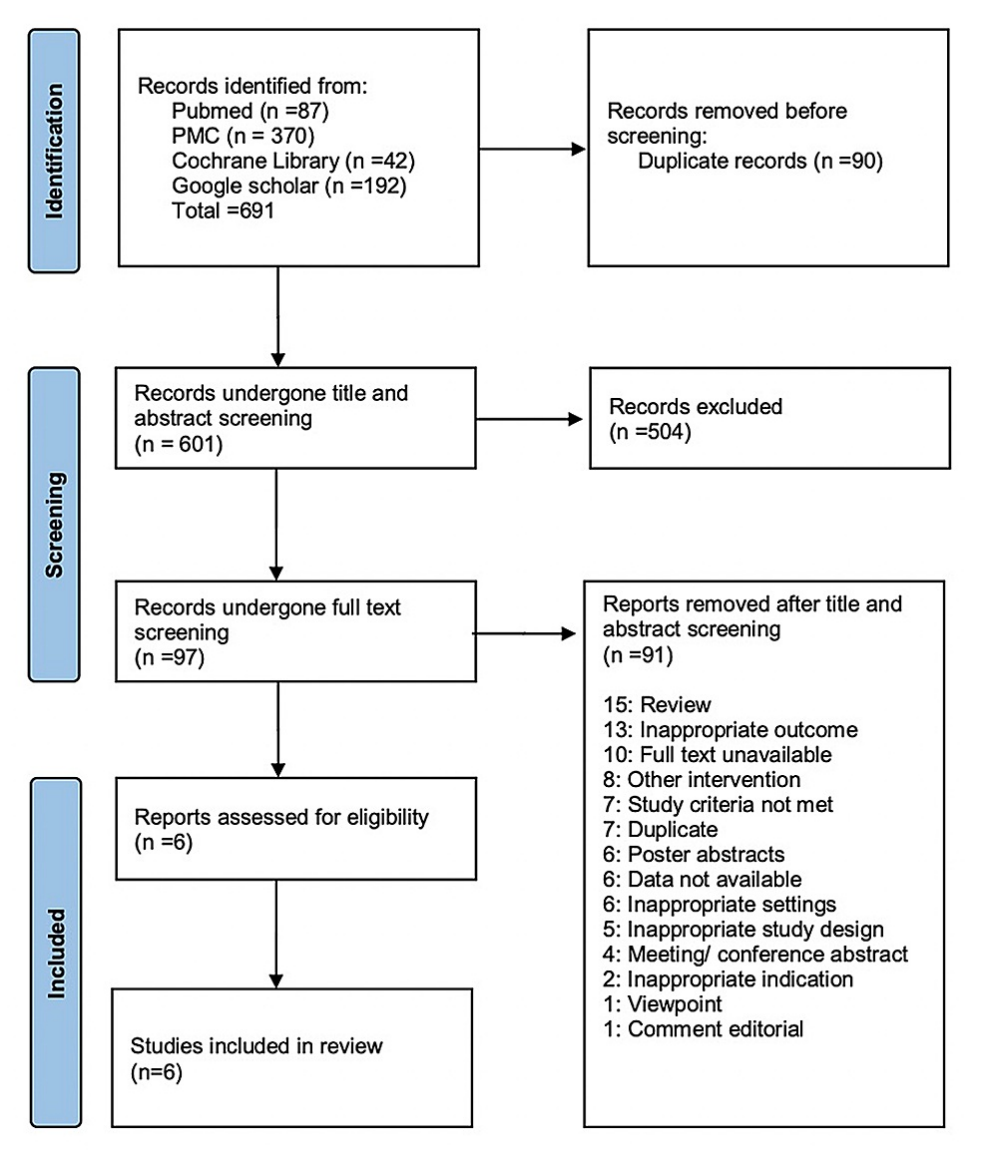
PRISMA flow chart of the studies included in the systematic review PRISMA: Preferred Reporting Items for Systematic Reviews and Meta-Analyses

On thorough literature search, we found 691 articles from various databases, of which we found 87 from PubMed, 370 from PMC, 42 from Cochrane Library, and 192 from Google Scholar. Ninety duplicates were found and hence were excluded. On screening the remaining articles, 504 articles were excluded during the title and abstract screening, as they were found to be of the wrong study design (literature reviews or opinion articles). The remaining 97 articles were further evaluated for inclusion in this systematic review by performing full-text screening. Ninety-one articles were excluded, as they did not meet the inclusion criteria during full-text screening. As a result, six studies were included for data extraction, which included one RCT and five non-RCTs. Figure 1 shows the PRISMA flow chart of the studies evaluated in this systematic review.

Included Studies and Quality Assessment

The RCT included in this systematic review is ATTR-ACT (Tafamidis in Transthyretin Cardiomyopathy Clinical Trial) [12]. ATTR-ACT is a phase III multicenter double-blinded study assessing 441 patients of cardiac amyloidosis, 106 with ATTRm (familial transthyretin-associated forms of amyloid) and 335 with ATTRwt (wild-type transthyretin-associated forms of amyloid) [12]. The five non-RCTs included in this systematic review are as follows: (i) Three phase II studies [13-15] assessing the efficacy of Tafamidis in 31, 21, and 21 patients; (ii) one multicenter observational study [16] assessing the efficacy of Tafamidis in 61 patients; (iii) one phase III study [17] assessing the efficacy of Tafamidis in 10 patients.

**Figure 2:**
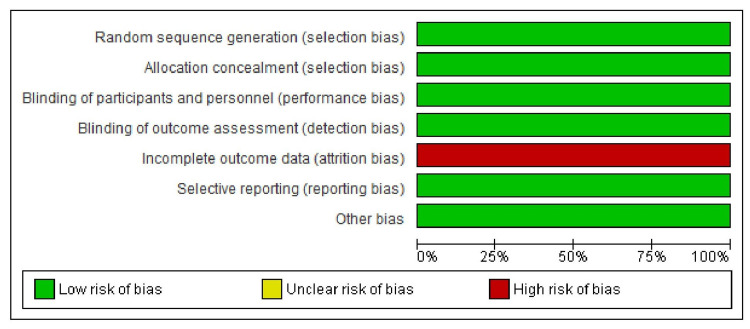
Risk of bias graph of ATTR-ACT trial assessed using the Cochrane risk of bias 2 tool ATTR-ACT: Tafamidis in Transthyretin Cardiomyopathy Clinical Trial

Figure 2 represents the risk of bias graph of the ATTR-ACT trial [12] assessed using the Cochrane RoB 2 tool. In the ATTR-ACT trial [12], randomization was carried out appropriately with adequate concealment of treatment allocation. Also, in the ATTR-ACT trial [12], care providers, participants, and outcome assessors were blinded to treatment allocation. However, there was significant missing data in both the Tafamidis and placebo groups. Thirty-four point forty-seven percent (34.47%) of participants’ data was missing in the Tafamidis group due to various reasons while the percentage was 51.98 in the placebo group. Hence, there was much imbalance between the two groups regarding the missing data in the ATTR-ACT trial. Table 1 represents the risk of bias assessment of five non-RCTs using the Downs and Black assessment checklist.

**Table 1:** Risk of bias assessment of non-RCTs using the Downs and Black assessment checklist RCT: randomized controlled trial; NA: not applicable; U: unable to determine

Question no.	Maurer et al. [13]	Damy et al. [14]	Merlini et al. [15]	Cortese et al. [16]	Yukio et al. [17]
i. Is the hypothesis/aim/objective of the study clearly described?	Yes	Yes	Yes	Yes	Yes
ii. Are the main outcomes to be measured clearly described in the introduction or methods sections?	Yes	Yes	Yes	No	Yes
iii. Are the characteristics of the patients included in the study clearly described?	Yes	Yes	Yes	Yes	Yes
iv. Are the interventions of interest clearly described?	Yes	Yes	Yes	No	Yes
v. Are the distributions of principal confounders in each group of patients to be compared clearly described?	Yes	Yes	Yes	Yes	Yes
vi. Are the main findings of the study clearly described?	Yes	Yes	Yes	No	Yes
vii. Does the study provide estimates of the random variability in the data for the main outcomes?	Yes	Yes	No	Yes	Yes
viii. Have all important adverse events that may be a consequence of the intervention been reported?	No	Yes	Yes	Yes	Yes
ix. Have the characteristics of patients lost to follow-up been described?	Yes	Yes	Yes	Yes	Yes
x. Have actual probability values been reported (e.g., 0.035 rather than <0.05) for the main outcomes except where the probability value is less than 0.001?	No	No	No	Yes	No
xi. Were all the subjects asked to participate in the study representative of the entire population from which they were recruited?	No	No	No	Yes	No
xii. Were those subjects who were prepared to participate representative of the entire population from which they were recruited?	No	Yes	No	No	No
xiii. Were the staff, places, and facilities where the patients were treated representative of the treatment the majority of patients receive?	No	No	Yes	Yes	No
xiv. Was an attempt made to blind study subjects to the intervention they have received?	No	No	No	No	No
xv. Was an attempt made to blind those measuring the main outcomes of the intervention?	No	No	No	No	No
xvi. If any of the results of the study were based on “data dredging,” was this made clear?	U	Yes	Yes	No	No
xvii. In trials and cohort studies, do the analyses adjust for different lengths of follow-up patients, or in case-cohort studies, is the time period between the intervention and outcome the same for cases and controls?	No	Yes	No	No	No
xviii. Were the statistical tests used to assess the main outcomes appropriate?	Yes	Yes	Yes	Yes	Yes
xix. Was compliance with the intervention(s) reliable?	U	U	U	U	U
xx. Were the main outcome measures used accurate (valid and reliable)?	Yes	Yes	Yes	Yes	Yes
xxi. Were the patients in different intervention groups (trials and cohort studies), or were the cases and controls (case-control studies) recruited from the same population?	NA	NA	NA	NA	NA
xxii. Were study subjects in different intervention groups (trials and cohort studies), or were the cases and controls (case-control studies) recruited over the same period of time?	NA	NA	NA	NA	NA
xxiii. Were study subjects randomized to intervention groups?	No	No	No	No	No
xxiv. Was the randomized intervention assignment concealed from both patients and health care staff until recruitment was complete and irrevocable?	No	No	No	No	No
xxv. Was there an adequate adjustment for confounding in the analyses from which the main findings were drawn?	No	No	No	No	No
xxvi. Were losses of patients to follow-up taken into account?	No	No	No	No	No

Table 2 shows the characteristics of participants of the studies included in the systematic review. The study characteristics of the RCT and non-RCTs included in the review are shown in Table 3. Table 4 shows the changes in echocardiographic parameters, cardiac biomarkers, quality of life, and percentage of TTR (transthyretin) stabilization achieved during the study.

**Table 2:** Characteristics of participants of the studies included in the systematic review SD: standard deviation; ATTR-ACT: Tafamidis in Transthyretin Cardiomyopathy Clinical Trial; * signifies median value (range); NA: not available; BMI: body mass index; TTR: transthyretin

Patient Characteristics	ATTR-ACT trial [12]		Maurer et al. [13]	Damy et al. [14]	Merlini et al. [15]	Cortese et al. [16]	Yukio et al. [17]
	Tafamidis (n=264)	Placebo (n=177)					
Age in years, Mean (SD)	74.5 (7.2)	74.1 (6.7)	76.7 (68.7-86.5)*	63.1 (9.9)	63.1 (9.9)	62 (11)	60.1 (13)
Sex, Male No. (%)	241 (91.3)	157 (88.7)	29 (93.5)	13 (61.9)	13 (61.9)	42 (69 )	7 (70)
Race No. (%)	White- 211 (79.9); Black- 37 (14.0); Asian- 13 (4.9); Other- 3 (1.1)	White-146 (82.5); Black- 26 (14.7); Asian- 5 (2.8); Other- 0	N/A	Afro-Caribbean- 1 (4.8); Asian- 1 (4.8); Caucasian- 19 (90.5)	N/A	N/A	N/A
Body mass index Mean (SD)	NA	NA	NA	NA	mBMI = 1,052.5 (206.7) kg/m^2^ x g/L; this mBMI is mean of 20 participants	mBMI = 978 (195) kg/m^2^ x g/L	BMI = 20.9 (3.1) kg/m^2^

**Table 3:** Study characteristics of the RCT and non-RCTs included in the review ATTR-ACT: Tafamidis in Transthyretin Cardiomyopathy Clinical Trial; ATTRwt: wild-type transthyretin-associated forms of amyloid; ATTRm: familial transthyretin-associated forms of amyloid; TTR: transthyretin; RCT: randomized controlled trial

Clinical Study Characteristics	ATTR-ACT trial [12]	Maurer et al. [13]	Damy et al. [14]	Merlini et al. [15]	Cortese et al. [16]	Yukio et al. [17]
Study design; Phase	Multicenter, double-blinded, placebo-controlled, parallel design, randomized; Phase III	Open level, single treatment arm; Phase II	Open level; Phase II	Open level, single treatment arm; Phase II	Multicenter observational study	Multicenter, single-arm, open-label; Phase III study
Participants No.	441	31	21	21	61	10
Duration of study	30 months	12 months	12 months	12 months	36 months	30 months (median treatment duration 713.5 days)
Treatment received	Participants received Tafamidis 80 mg, Tafamidis 20 mg, or matching placebo once daily assigned in the ratio of 2:1:2	20 mg Tafamidis once daily	20 mg Tafamidis once daily (in a soft gelatin capsule)	20 mg Tafamidis once daily	20 mg Tafamidis once daily	Once-daily oral dose of Tafamidis meglumine 20 mg
Control group	Placebo	None	None	None	None	None
Study Population	Transthyretin amyloid cardiomyopathy (ATTRwt or ATTRm) confirmed patients; 18 to 90 years	Men and postmenopausal women $\geq$ 40 years with V122I or wild-type transthyretin cardiomyopathy diagnosed via cardiac biopsy or noncardiac biopsy	Symptomatic, biopsy-confirmed TTR amyloid polyneuropathy; 18 to 75 years; men and non-pregnant women	Symptomatic, biopsy-confirmed TTR amyloid polyneuropathy due to a known pathogenic TTR mutation other than Val30Met or Val122Ile; 18 to 75 years; men and non-pregnant women	Women and men aged $\geq$ 18 affected by symptomatic ATTR-related neuropathy; patients starting treatment with Tafamidis meglumine (20 mg once daily); written informed consent to participate in the study	Men and women aged 20 to 75 years with documented amyloid deposition by biopsy and Val30Met or other TTR mutation (non-Val30Met)

**Table 4:** Changes in echocardiographic parameters, cardiac biomarkers, quality of life, and % of transthyretin stabilization achieved during the study TTR: transthyretin; ATTR-ACT: Tafamidis in Transthyretin Cardiomyopathy Clinical Trial; NA: not available; IVS: interventricular septal thickness; LV: left ventricle; LVEF: left ventricular ejection fraction; M: month; SD: standard deviation; NT-proBNP: N-terminal pro-B-type natriuretic peptide; AE: Adverse Event; SAEs: serious adverse events; TIA: transient ischemic Attack; AV: Atrioventricular; HR: hazard ratio; CI: confidence interval; AL: amyloid light-chain; NYHA: New York Heart Association; KCCQ-OS: Kansas city cardiomyopathy questionnaire-overall summary; TQOL: total quality of life; Norfolk QOL-DN: Norfolk quality of life-diabetic neuropathy questionnaire

Results	ATTR-ACT trial [12]	Maurer et al. [13]	Damy et al. [14]	Merlini et al. [15]	Cortese et al. [16]	Yukio et al. [17]
% of TTR stabilization achieved	NA	96.8% and 89.3% achieved TTR stabilization after 6 weeks and 12 months respectively.	NA	100% TTR stabilization at 6 and 12 months	NA	100% TTR stabilization at week 8, and 26 whereas 90% and 80% on 52 and 78 weeks
Changes in Echocardiographic parameters during the study period	The difference of change in % of circumferential and radial mid-global strain from baseline to month 30 between Tafamidis and placebo group was statistically significant; other parameters like left ventricular end-diastolic interventricular septal wall thickness, left ventricular posterior wall thickness, left ventricular ejection fraction did not achieve statistical significance.	Wall thickness and pressure has no clinically relevant changes seen on the echocardiogram	12/19 Patients has new-onset echocardiographic abnormalities; 2 patients with normal IVS at baseline, LV wall thickness remained below > 12 mm; 4/12 patients demonstrated ≥ 2 mm increase in IVS thickness; 6 had more than 10% increase in LV mass; 7 has stable LV mass; 1 patient has more than 10% decreased; none developed LV systolic dysfunction; LVEF deteriorated by more than 10% in 2/18 patients; no significant changes in left ventricular filling pressure	Echocardiographic parameters remained relatively stable from baseline to month 12 with no clinically relevant changes	In 34 patients with cardiac involvement at baseline Mean IVS increased from baseline by 0.6 ± 1.6 mm at M12 and 1.05 ± 2.0 mm at M24; 15% of patients showed echocardiographic evidence of cardiac disease progression	Mean (SD) change in septum diastole thickness from baseline was decreased by −1.3 (SD= 2.7) mm at Week 52 and −3.7 (SD=3.7) mm at the end of the study; the mean increase in stroke volume was 1.1 (SD=5.0) mL at week 52 and 6.0 (SD=13.0) mL at the end of the study
Changes in the level of cardiac biomarkers during the study period	A statistically significant smaller increase in the NT-proBNP level among participants of Tafamidis group than placebo at months 12 and 30	Median NT-proBNP concentration was elevated at baseline; the LS mean increase of NT-proBNP was nonsignificant from baseline to M12; on assessing Troponin I and troponin T levels after 3 months showed elevation	No clinically relevant changes in mean NT-proBNP concentration and mean Troponin I concentration	Baseline troponin I concentration remained stable throughout the study; the pro-BNP level remained increased with no clinically relevant changes over time (mean increase of pro-BNP concentration 228.4 at month 6 and 306.6 at month 12)	3 Patients had NT-proBNP increase by ≥30%	NA
Adverse events	The occurrence of adverse events was similar both in Tafamidis and Placebo groups; Treatment-emergent adverse events were mild to moderate; Permanent drug discontinuation rate due to adverse events was less in Tafamidis group; Common adverse events were blood and lymphatic disorders, cardiac disorders (cardiac failure and atrial fibrillation), eye disorders, gastrointestinal disorders (nausea, diarrhea, constipation)	All 31 patients had ≥ 1 AE; SAEs in 13/31; Cardiac failure in 8/31; Atrial fibrillation in 3/31; Fall in 3/31; Syncope in 2/31	3 discontinued early ( one due to TIA and 2 to undergo liver transplant); no life-threatening AEs or deaths; 8 patients had serious treatment-emergent AEs, 5 experienced fall; 3 experienced one serious cardiovascular adverse event ( 1 coronary artery stenosis, one TIA, and 1 AV block)	81% of participants experienced at least one AE, most common was falls; 4 SAEs occurred that were considered related to treatment	13% of patients reported AE; no discontinuation of treatment due to Tafamidis-related AEs	85 all-cause AEs were reported in 10 patients and 2 AEs in 2 patients were treated as treatment-related AEs; severe AEs occurred in 3 patients
All-cause mortality	Lower in Tafamidis vs placebo [78/264 (29.5%) vs 76/177 (49.9%); HR=0.75, 95% CI= 0.51 to 0.96]	2 patients died during the study period: 1 patient died from complications resulting from AL amyloidosis and the other died of hemorrhagic shock after a fall	NA	NA	NA	NA
Cardiovascular related hospitalization	The frequency of cardiovascular-related hospitalizations was lower in the Tafamidis group in comparison to placebo [0.48 per year vs 0.70 per year; relative risk ratio of 0.68, 95%CI = 0.56 to 0.81]	7 patients were hospitalized because of cardiovascular events	NA	NA	12 patients progressed to higher NYHA failure class; one required pacemaker implantation after 18 months of treatment	NA
Ambulatory changes	NA	NA	NA	NA	NA	Walking was preserved in half of the patients over the 1.5-year treatment duration with Tafamidis
Quality of Life Assessments during the study duration	Lower rate of decline in the distance for the 6-minute walk test (P<0.001) and KCCQ-OS score (P<0.001).	6-minute walk test: the mean distance walked during 6-minute was decreased by 8.9 meters from baseline to month 12. Several measures of health-related quality of life: NHYA classification maintained in 71.4% and no patient deteriorated by ≥2 class or dropped to NHYA classification IV; overall patients demonstrated a preserved health-related quality of life	NA	Mean changes in TQOL score were -4.3 at month 6 and 0.1 at month 12.	NA	Mean change in TQOL score assessed by Norfolk QOL-DN from baseline was 11.8 at week 26, 9.1 at week 52, and 10.8 at week 78

TTR Stabilization

TTR stabilization was assessed in three non-RCTs [13,15,17], which compared baseline data with data at the end of the study. In Merlini et al. [15], 100% of participants demonstrated TTR stabilization throughout the study period. At week 8 and week 26, TTR stabilization was maintained in 100% of participants in the Yukio et al. [17], but at weeks 52 and 78, it was maintained in 90% and 80% participants, respectively. In Yukio et al. [17], two participants had missing data regarding TTR stabilization at week 72. In the Maurer et al. [13], 96.8% of participants with wild-type TTR-CM had TTR stabilization at week 6, whereas only 89.3% of participants maintained TTR stabilization at month 12. A randomized double-blinded trial that studied Tafamidis in TTR-FAP patients demonstrated TTR stabilization in 98% of patients in the Tafamidis group while none of the participants in the control group achieved TTR stabilization during the 18-month study period [18]. This explains the TTR stabilizing property of Tafamidis.

Echocardiographic Changes

Changes in findings of echocardiography were assessed in the ATTR-ACT trial [12] and five non-RCTs [13-17]. In three studies (Maurer et al. [13], Damy et al. [14], and Merlini et al. [15]), there were no clinically relevant changes from baseline. Damy et al. [14] showed the occurrence of new-onset echocardiographic findings in 12/19 patients while Cortese et al. [16] demonstrated a 15% progression of cardiac disease based on echocardiography. There were variable findings regarding interventricular septal (IVS) thickness.

Cardiac Biomarkers

Cardiac biomarkers were assessed in the ATTR-ACT trial [12] and four non-RCT studies [13-16]. NT-proBNP (N-terminal pro-B-type natriuretic peptide) level was evaluated in the ATTR-ACT trial [12] and four non-RCT studies [13-16], whereas Troponin I was evaluated in three non-RCT studies [13-15]. The NT-proBNP level was found to be stable in two studies (Damy et al. [14] and Merlini et al. [15]) while Cortese et al. [16] demonstrated an increase in NT-proBNP levels while in Maurer et al. [13], the increase was insignificant. Out of three studies evaluating Troponin I levels, the levels remained stable in two studies [12-13] and were found to be increased in one study [13].

Safety

The safety profile of Tafamidis was evaluated in the ATTR-ACT trial [12] and five non-RCTs [13-17]. In the ATTR-ACT trial, there were no significant differences in adverse events in the Tafamidis and placebo groups. All adverse events that appeared during the treatment period were mild to moderate in severity, and withdrawal of treatment due to adverse events was less in the Tafamidis group in comparison to placebo. In Maurer et al. [13], all patients with wild-type TTR-CM experienced at least one adverse event. Most of them had symptoms of heart failure while 22.6% (7/31) of participants experienced diarrhea and 16.1% (5/31) had weight gain. Serious adverse events occurred in 41.9% of participants (13/31), which included heart failure, atrial fibrillation, syncope, and pacemaker insertion. In the Maurer et al. study [13], two patients died and seven patients were hospitalized for cardiovascular events (CVEs) during the treatment period. In Damy et al. [14], the overall safety profile of Tafamidis was good. Only 38.1% (8/21) of patients developed serious adverse events. In Merlini et al. [15], 81% of participants (17/21) developed at least one adverse event during the study period. However, in Merlini et al. [15], only four patients had serious adverse events and no mortality was reported during the study period. In Cortese et al. [16], 13% of patients (8/61) experienced adverse events, of which three had serious adverse events. In the Yukio et al. [17] study, all 10 patients experienced at least one adverse event, but serious adverse events occurred only in three patients.

All-Cause Mortality and Cardiovascular Related Hospitalization

All-cause mortality and cardiovascular-related hospitalization were assessed in one RCT [12], which demonstrated a decrease in all-cause mortality in patients under Tafamidis vs. placebo. Maurer et al. [13] demonstrated minimal deaths and hospitalization in patients treated with Tafamidis. Cortese et al. [16] showed the progression of 12 patients to a higher NYHA (New York Heart Association) class, and one patient required pacemaker implantation.

Quality of Life

Quality of life was assessed in the ATTR-ACT trial [12] and three non-RCTs (Maurer et al. [13], Merlini et al. [15], and Yukio et al. [17]). The ATTR-ACT trial [12] and Maurer et al. [13] showed a lower rate of decline in the six-minute walk test. Yukio et al. [17] and Merlini et al. [15] demonstrated that quality of life was maintained throughout treatment with Tafamidis. Yukio et al. [17] also showed maintenance of ambulatory status in 50% of patients during their treatment.

Modified BMI

Modified BMI (mBMI) was assessed in three non-RCTs (Merlini et al. [15], Cortese et al. [16], and Yukio et al. [17]) where no changes were seen in Cortese et al. [16], and modified BMI increase was demonstrated in Yukio et al. [17]. Merlini et al. [15] showed an initial decrease in BMI followed by an increase in modified BMI.

Discussion

TTR tetramer dissociation has been the rate-limiting step in TTR amyloid formation. Tafamidis is one of the TTR stabilizing compounds that have been studied well. It is essential to give evidence to the scientific community relating the different effects of Tafamidis on clinical parameters. This will help establish the role of Tafamidis in transthyretin amyloid cardiomyopathy.

In Maurer et al. [13], 30 participants with wild-type TTR-CM had increased interventricular septum and left ventricular posterior wall thickness. Out of 30 wild TTR-CM patients in the Maurer et al. study [13], 73.7% of patients had elevated left ventricular filling pressure while 64% of participants had elevated right ventricular filling pressure, and 43.3% had an ejection fraction of <50%. In the Maurer et al. study [13], throughout the treatment period with Tafamidis, 30 patients with wild-type TTR-CM did not have clinically relevant changes in the above parameters. In Damy et al. [14], participants had several echocardiographic abnormalities at baseline like LV posterior wall thickness, interventricular septal thickness, and isovolumetric relaxation time. However, during the 12-month treatment period with Tafamidis, the average values for these echocardiographic parameters remained stable. In Merlini et al. [15], echocardiographic parameters like posterior LV wall thickness, interventricular septal thickness, left atrial diameter, left ventricular mass, and LV ejection fraction were assessed at baseline and month 12 of the study. During the treatment period, participants of Merlini et al. [15] had relatively stable echocardiographic parameters over 12 months of treatment with Tafamidis without any clinically significant changes. In Cortese et al. [16], 34 patients out of 61 had cardiac involvement, of which mean interventricular septum thickness increased by 0.6 $\pm$ 1.6 mm at month 12 and 1.05 $\pm$ 2 mm at month 24 but only five patients had cardiac disease progression. During the study duration of Cortese et al. [16], out of 23 patients without cardiac involvement, eight developed cardiomyopathy, and 12 patients progressed to a higher NYHA heart failure class. In Yukio et al. [17], interventricular septum diastolic thickness decreased at the end of the study while stroke volume increased at the end of the study.

Measurement of myocardial strain is a method to quantify left ventricular function by using speckle-tracking echocardiography. There has been a system to measure strain along three cardiac axes: circumferential, radial, and longitudinal [19]. The three-axis system will allow measuring the shortening and elongation of the myocardium through the cardiac cycle in three directions in reference to the measurements during the time of QRS-complex [19]. Among the measurements in three cardiac axes, global longitudinal strain (GLS) is more sensitive and important for the measurement of systolic function than left ventricular ejection fraction [19]. GLS has the power to uncover even subtle or subclinical LV dysfunction in cardiomyopathies. In the ATTR-ACT trial [12], the difference of change in percentage of circumferential and radial mid-global strain from baseline to month 30 between the Tafamidis and placebo groups was statistically significant. However, the difference of change in percentage of global longitudinal strain from baseline to month 30 between the Tafamidis and placebo groups was not statistically significant. Another echocardiographic parameter that gained statistical significance was the difference in change in volume of left ventricular stroke volume from baseline to month 30 between the Tafamidis and placebo groups (LS mean difference 6.28 ml; 95% CI 1.96 to 10.59). The decrease in left ventricular stroke volume was less (5.38 ml) in the Tafamidis group in comparison to the placebo (11.66 ml). Also, other parameters like left ventricular end-diastolic interventricular septal wall thickness, left ventricular posterior wall thickness, and left ventricular ejection fraction did not achieve statistical significance.

Therefore, there was the stability of different echocardiographic parameters in single-arm non-RCT studies. In the ATTR-ACT trial [12], the scenario is different. Although there was a statistically significant difference in the change in stroke volume at the end of the trial between the Tafamidis and placebo groups, with a lower decrease in the Tafamidis group, there was no statistically significant difference of changes noted in other echocardiographic parameters during the follow-up period.

In Damy et al. [14], 76.2% of patients had ECG abnormalities at baseline. During the study period of 12 months, 42.9% of patients developed new-onset ECG abnormalities o,f which arrhythmias were most common. In the Maurer et al. [13] study, 100% of participants had ECG abnormalities while 80% of participants had abnormalities during Holter monitoring. During the study period, ECG abnormalities associated with treatment were low. In Merlini et al. [15], 44.4% of participants developed a treatment-associated ECG abnormality. In patients with amyloidosis, ECG and Holter abnormalities represent cardiac autonomic dysfunction [14]. In the above studies, we can find treatment-associated ECG abnormalities to be mild to moderate. Further, the ECG and Holter abnormalities that existed at baseline could be co-related with a cardiac abnormality at baseline. However, to prove that Tafamidis improves and prevents cardiac autonomic dysfunction, a large prospective cohort study and a randomized clinical trial are needed.

NT-proBNP indicates raised intraventricular pressures in congestive heart failure and carries a significant prognostic value for mortality and cardiac disease progression [20]. Also, some evidence suggests NT-proBNP be an important marker of cardiac hypertrophy and LV function in hereditary TTR amyloidosis patients [21-22]. In the Maurer et al. [13] study, the median NT-proBNP of participants was raised. Although there were many variations in NT-proBNP values among participants, there was no significant increase in NT-proBNP at the end of the study in comparison to baseline. In the Damy et al. [14] and Merlini et al. [15] studies, mean NT-proBNP was raised at the start of the study, but no significant elevation was noted during the study period with Tafamidis. In the Cortese et al. study [16], 42% of patients had altered NT-proBNP level, but during the treatment period with Tafamidis, only three of 23 patients without heart involvement had an increase of NT-proBNP by 30%. In the ATTR-ACT trial [12], while exploring other endpoints, we found a statistically significant smaller increase in the NT-proBNP level among participants of the Tafamidis group than placebo at months 12 and 30. This is in contrast to non-RCT studies that show either a decrease or stable NT-proBNP level among the Tafamidis group. Hence, there are variations in the change in the level of NT-proBNP marker between single-arm non-RCTs and RCT.

In the Damy et al. [14] and Merlini et al. [15] studies, the mean Troponin I level of participants was normal before the start of the study and remained stable during the study period. This is consistent with a previous study that reports NT-proBNP as a more sensitive marker of cardiomyopathy in patients with TTR amyloidosis than Troponin I [21]. The stability in the value of Troponin I during the study period probably could be attributed to Tafamidis in halting the progression of cardiac disease in TTR-CM patients. In the Maurer et al. [13] study, 100% of participants had raised Troponin I at baseline. Following an initial drop in the concentration of Troponin I at month 3, Troponin I level increased at month 12 in comparison to baseline. However, the increase in Troponin I was comparable to the TRACS (Transthyretin Amyloidosis Cardiac Study) study where a group of untreated TTR-CM patients had a similar elevation in Troponin I [23].

ATTR-ACT [12] is a placebo-controlled randomized multicenter trial that studied the efficacy of Tafamidis in transthyretin amyloid cardiomyopathy patients. In the ATTR-ACT trial [12], the all-cause mortality was lower in the Tafamidis group in comparison to placebo [HR=0.70, 95% CI, 0.51 to 0.96]. Previous case series have reported a one-year mortality rate of 14% to 23% in wild-type TTR-CM patients. Hence, the reduction in all-cause mortality associated with Tafamidis is significant when we consider the natural history of the disease. Also, in the ATTR-ACT trial [12], the Tafamidis group had a lower rate of cardiovascular-related hospitalizations in comparison to placebo (relative risk ratio=0.68; 95% CI, 0.56 to 0.81).

In the ATTR-ACT trial, the distance covered during the six-minute walk test was prevented from further deteriorating. The Kansas City Cardiomyopathy Questionnaire-Overall Summary (KCCQ-OS) scoring system is the measure of functional capacity and quality of life in cardiomyopathy patients. Scores in the lower range in the KCCQ-OS scoring system denote a low quality of life. In the ATTR-ACT trial [12], Tafamidis slowed the lowering of the KCCQ-OS score. In the Maurer et al. [13] study, most of the patients maintained the distance achieved during the six-min walk test. Also, in Maurer et al. [13], 71.4% of patients had stable NYHA classification with no patient deteriorating two classes or reaching the IV NYHA classification. In Maurer et al. [13], the overall quality of life of participants was maintained on different scoring systems like global patient assessment, Kansas City Cardiomyopathy Questionnaire, and Short Form 36 scoring systems. In the Merlini et al. [15] study, Norfolk Quality of life-Diabetic Neuropathy was used to assess the quality of life where higher scores denote a worse quality of life. The quality of life scoring was maintained during the study period in Merlini et al. [15] study. In the Yukio et al. [17] study, during the treatment duration of 1.5 years, walking was preserved in 50% of patients. Also, in the Yukio et al. [17] study, change in the total quality of life from baseline was stable at weeks 26, 52, and 78.

Hence, considering the findings of non-RCTs, we can associate Tafamidis with reducing cardiac disease progression in patients with TTR-CM. Analyzing the echocardiographic parameters, cardiac biomarkers (NT-ProBNP, Troponin I), ECG, and quality of life measures in five non-RCTs, the measurement parameters were stable during the treatment period. But in the absence of a control group in five non-RCTs, it is challenging to predict the changes in the control group. However, the change in parameters being stable in all five non-RCTs rules out the findings to be due to chance alone. In the TRACS study, 18 wild-type and 11 V122I patients with TTR-CM were followed without treatment for 12 months. In the TRACS study, the ejection fraction of participants as measured by echocardiography decreased by -8% to 50.5%, whereas the NT-proBNP level was raised by 1487 to 6268 pg/ml. Therefore, correlating the findings of the TRACS study [23] with the findings of five non-RCTs, we can positively associate Tafamidis use and stable cardiac parameters during the study period. Also, we can say the drug has a good safety profile by analyzing the safety profile of Tafamidis in the ATTR-ACT trial [12] and five non-RCTs [13-17].

Thus, the ATTR-ACT trial [12] shows a decrease in all-cause mortality and cardiovascular-related hospitalizations in the Tafamidis group in comparison to placebo. But assessing the progression of disease in terms of cardiac parameters, we get variances in findings in the ATTR-ACT trial in comparison to single-arm non-RCTs included in this review. Hence, further randomized clinical trials that study different echocardiographic parameters are needed to provide robust evidence of Tafamidis in reducing cardiac disease progression. Currently, there are few ongoing clinical trials of Tafamidis. NCT04814186 is a phase 4 clinical trial of Tafamidis registered with ClinicalTrials.gov on March 24, 2021, to evaluate the safety and efficacy of once-daily treatment in Chinese participants with TTR-CM, which is expected to be completed by November 24, 2023. NCT02791230 is an ongoing phase 3 clinical trial registered with ClinicalTrials.gov on June 6, 2016, to study the safety of once-daily treatment with Tafamidis meglumine in TTR-CM patients. The findings of these clinical trials will further provide additional evidence of the safety and efficacy of Tafamidis in TTR-CM patients.

## Conclusions

Tafamidis is a TTR-stabilizing drug that has been associated with reducing cardiovascular events and all-cause mortality in patients with TTR-CM disease. Analyzing the non-RCTs findings, we can associate the drug with stabilizing cardiac parameters and halting the cardiac disease progression. However, analyzing the echocardiographic parameters and biomarkers in the ATTR-ACT trial, we get variances in findings in comparison to single-arm non-RCTs. On overall analysis, we can say for sure that Tafamidis is a safe and effective drug for TTR-CM patients. However, the results of future RCTs of Tafamidis can provide robust evidence on the role of Tafamidis in halting cardiac disease progression in transthyretin amyloid cardiomyopathy.

## Appendices

Search strategy

PubMed: 87

(((("amyloid cardiomyopath*") OR ("amyloid cardiomyopath*"[Title/Abstract])) OR ("amyloid cardiomyopath*"[MeSH Terms]) OR ("Cardiac amyloid*")) OR (("Cardiac amyloid*")[Title/Abstract])) OR (("Cardiac amyloid*")[MeSH Terms])

PubMed Central (PMC): 370

((("tafamidis"[Supplementary Concept] OR "tafamidis"[All Fields]) OR Tafamidis[Title]) OR Tafamidis[Abstract]) AND ((("amyloid cardiomyopathy"[All Fields] OR "amyloid cardiomyopathy*"[Abstract]) OR "amyloid cardiomyopathy*"[Title]) OR (("cardiac amyloid*"[All Fields] OR "cardiac amyloid*"[Abstract]) OR "cardiac amyloid*"[Title]))

Cochrane Library: 42

Date Run: 06/27/2021

Comment:

ID                  Search Hits

#1                 Tafamidis   103

#2                 amyloid cardiomyopathy   98

#3                 #1 and #2         42

Google Scholar: 192

Tafamidis AND ("amyloid cardiomyopathy" OR "cardiac amyloidosis")

Total no. of articles: 691
